# A rapid review exploring nurse‐led memory clinics

**DOI:** 10.1002/nop2.688

**Published:** 2020-11-18

**Authors:** Kerrie E. Luck, Shelley Doucet

**Affiliations:** ^1^ University of New Brunswick Saint John NB Canada; ^2^ Dalhousie Medicine New Brunswick Saint John NB Canada

**Keywords:** advanced nursing practice, advanced practice nurse, Alzheimer's, dementia, memory clinic, non‐medical prescriber, nurse, nurse practitioner, nurse‐led, rapid review

## Abstract

**Aims:**

To systematically explore the structures, functions, outcomes, roles and nursing credentials of memory clinics where nurses autonomously lead diagnosis and postdiagnostic care.

**Design:**

A systematic rapid review was conducted.

**Data sources:**

MEDLINE (Ovid), CINAHL Full‐Text (EBSCO) and EMBASE were systematically searched in December 2019 with no timeframe limitations imposed.

**Review Methods:**

The modified PRISMA checklist was used as a guide to facilitate the review. Articles identified were screened and assessed for inclusion criteria, and screening of reference lists of included studies was also completed.

**Results:**

Six articles, published between 2011–2019, including two case studies, two descriptive reports, one qualitative study and one programme evaluation were included in the review. Nurse‐led memory clinics were situated in community centres, on university campuses, hospitals and in general practitioners' offices. The services offered included assessment, diagnosis and treatment/postdiagnostic care. Nurse credentials included advanced practice nurses and a community psychiatric nurse who was a non‐medical prescriber. Overall, there was low quantity and quality of evidence to evaluate outcomes.

## 1 INTRODUCTION

An estimated 47 million people globally have dementia, with approximately 10 million new cases reported annually (Prince et al., 2015; WHO, 2015). With an ageing population, this number is predicted to reach 132 million by 2050 (WHO, 2017). The World Health Organization (2017) predicts 5%–8% of the population over the age of 60 have dementia, which is one of the main causes of functional decline among this demographic worldwide. However, it is estimated in higher income countries that up to 50% of individuals with dementia still go undiagnosed due to stigma, false beliefs about the disease (e.g. memory problems are a normal part of ageing, nothing can be done), lack of medical education in primary care (i.e. medical doctors have reported they do not have the training they need) and accessibility to diagnostic services (Prince et al., 2011).

While there is currently no cure for dementia, early diagnosis is critical to optimize timely access to care and to promote the quality of life of those living with this disease (Prince et al., 2011; WHO, 2017). With an ageing population and increasing demographic, the need for early diagnosis and access to postdiagnostic care is in great demand, now more than ever. Traditionally, physician‐run memory clinics have been used to try and meet this need by providing early assessment, diagnosis and treatment and by facilitating dementia follow‐up care (e.g. providing resources and information; teaching; coordinating care) (Jolley & Moniz‐Cook, 2009); however, with the growing demands, alternatives or variations to this approach are necessary. Nurse‐led memory clinics may be a complementary model to help address this growing need of care for those living with or at risk for dementia and their caregivers.

### 1.1 Background

Memory clinics, led by specialist physicians and run out of academic hospitals as an outpatient‐based service, were introduced in the 1980s (Jolley et al., 2006; Van der Cammen er al., 1987). The initial aim of these clinics was for research purposes; however, the memory clinic model has developed over the years including variations in settings, team members, referral processes, patient characteristics and services. These changes have better addressed the needs of individuals living with dementia and their caregivers including timely assessment, diagnosis and follow‐up care (Hansen et al., 2017; Jolley et al., 2006; Minstrell et al., 2015). While there may be differences among clinics, most memory clinics have some form of multidisciplinary team structure led by a specialist physician to provide specialized assessment and early intervention, including neuropsychological testing, neuroimaging and psychosocial evaluations (Jolley & Moniz‐Cook, 2009; Lindesay et al., 2002; Ramakers & Verhey, 2011; Woodward & Woodward, 2009).

The increasing prevalence of dementia cases requiring diagnostic services, in combination with financial constraints, rising expectations among patients and a limited workforce (including a short supply of physicians), places a huge burden on our current healthcare systems (Hansen et al., 2017; Jolley et al., 2006; Laurant et al., 2005; Minstrell et al., 2015). Reves et al. (2018) suggest innovative models for dementia diagnosis and care need to be explored to improve outcomes, while being cost‐effective and efficient. Nurse‐led memory clinics (NLMC), sometimes referred to as nurse practitioner‐led clinics, have been suggested as an alternative to the traditional memory clinic model to improve the need for access to dementia diagnosis and care (Hansen et al., 2017; Minstrell et al., 2015). It should be noted that in this instance, a “nurse‐led memory clinic” does not necessarily mean a nurse working in isolation, but rather being a lead for diagnostic and postdiagnostic care for clients with dementia, in a similar fashion as the more traditional “physician‐ run” clinics previously mentioned. Various forms of nurse‐led clinics (i.e. being led by Registered Nurses, specialist nurses and/or nurse practitioners, with varying degrees of autonomy and responsibility) have been shown to provide quality care (Carey & Courtenay, 2007; Hansen et al., 2017; Lewis et al., 2009; Minstrell et al., 2015; Morgan et al., 2013); have a positive impact on patient outcomes (Carey & Courtenay, 2007; Hansen et al., 2017; McLoughney et al., 2007; Minstrell et al., 2015; Morgan et al., 2013); and use less financial and human resources (Carey & Courtenay, 2007; Lewis et al., 2009).

NLMC are typically led by Advanced Practice Nurses (APNs), which are nurses with advanced nursing education and clinical skill sets (International Advanced Practice Nursing, 2013). The International Council of Nurses (2020) defines an APN as a “registered nurse who has acquired the expert knowledge base, complex decision‐making skills and clinical competencies for expanded practice, the characteristics of which are shaped by the context and/or country where s/he is credentialed to practice. A master's degree is recommended for entry level” (definition section). Each country's regulatory body identifies various categories under the APN umbrella according to scope of practice, for example: nurse practitioner, advanced nurse practitioner or clinical nurse specialist. While some categories of APNs are not able to both diagnose and/or prescribe, other categories (i.e. nurse practitioner) do have the ability to autonomously diagnose, treat and provide ongoing care to patients. Page et al. (2008) demonstrated specialist nurses can accurately diagnose dementia in a memory clinic and suggest they should have a bigger role in memory clinics to improve access to diagnosis and treatment.

While nurse‐led memory clinics are a novel approach that appears to have merit, little is known about nurses in this type of role in this setting (Stirling et al., 2012, 2016). To date, no systematic review has been conducted to consolidate the current approaches and practices of nurse‐led memory clinics where the nurse autonomously leads both the diagnosis and postdiagnostic care for individuals with dementia. One systematic literature review did explore nurse prescribing in memory services (Emrich‐Mills et al., 2019); however, most of the articles reviewed were for supplementary prescribing after a diagnosis was made by a physician. While nurse prescribing has the potential to improve efficiencies (e.g. timey access to reliable diagnosis and treatment; cost‐effective care; Emrich‐Mills et al., 2019; McInally, 2015), our understanding of nurses comprehensively leading the care of individuals with dementia in a memory clinic, from diagnosis through to postdiagnostic care, is limited.

## 2 THE REVIEW

### 2.1 Aims

The aim of this rapid review was to systematically explore the structures, functions and outcomes of nurse‐led memory clinics, and the nursing roles and credentials of nurses leading memory clinics to inform nursing practice; enlighten discussions about interventions and innovations to improve the diagnosis and treatment of dementia; and identify areas for future research. This was accomplished through the systematic exploration of the questions: (a) *what are the structures, functions and outcomes of nurse‐led memory clinics?*; and (b) *what are the roles and credentials of nurses leading memory clinics?* For the purposes of this review, “nurse‐led memory clinic” was defined as a memory clinic/service or specialized geriatric clinic/service, with a nurse working autonomously leading both the diagnosis and postdiagnostic care for clients with dementia.

### 2.2 Design

A rapid review approach was chosen given the limited time and resources available to the authors to produce consolidated evidence to inform local practice, policy and research discussions on NLMC, while maintaining quality and credibility (Haby et al., 2016; O'Leary et al., 2017). The PRISMA (Preferred Reporting Items for Systematic Reviews and Meta‐Analyses) checklist (Moher et al., 2009) was modified (including the risk of bias checklist item) and used as a guide to facilitate a rapid review of the published research on this topic. Review modifications, which were aligned with rapid review methodology, included the following: (a) a targeted research question; (b) fewer searched databases; (c) reduced time frame; (d) exclusion of grey literature; and (e) use of one reviewer (Haby et al., 2016). According to Stevens et al. (2018), while PRISMA is a reporting guideline intended for systematic reviews and meta‐analysis, many published rapid reviews have used this as a guide due to the lack of rapid review specific guidelines. These authors are currently addressing this gap through developing a protocol to develop PRISMA‐RR for rapid reviews. Rapid reviews have been demonstrated as a practical approach to informing healthcare decisions, nursing policy and nursing practice (O'Leary et al., 2017).

### 2.3 Search methods

An initial limited search of MEDLINE (Ovid), CINAHL Full‐Text (EBSCO) and EMBASE (Elsevier) was undertaken to identify search terms and articles on this topic. In collaboration with a librarian (AG), a full search strategy was developed by using text words and index terms gathered from relevant articles. Each identified search term was tested in each database (i.e. MEDLINE, CINAHL, EMBASE), and only those producing unique results were included in the final strategy. The search strategy was peer reviewed by a second librarian (RW) using the Peer Review of Electronic Search Strategies (PRESS) guidelines (CADTH, 2016). Since rapid reviews use fewer search databases (Haby et al., 2016), these three databases were identified in partnership with two health‐science librarians (AG and RW) to ensure they would produce the best search result on the topic of interest. Full search strategies for all databases, conducted in December 2019, are available in Table 1. The reference list of all studies selected for critical appraisal was also searched for additional studies.

**TABLE 1:** Database search strategy

No.	Query	Results
CINAHL Full‐Text (EBSCO)—Search conducted December 2019		
1	(MH "Nurse Practitioners+")	20,640
2	(MH "Advanced Practice Nurses+")	34,749
3	(MH "Advanced Nursing Practice+")	13,668
4	TI “nurse‐led” OR AB “nurse‐led”	3,919
5	TI “nurse practitioner*” OR AB “nurse practitioner*”	12,886
6	TI “advanced practice* nurs*” OR AB “advanced practice* nurs*“	3,807
7	TI "nurs* prescri*" OR AB "nurs* prescri*"	1,592
8	TI NP OR AB NP	4,981
9	S1 OR S2 OR S3 OR S4 OR S5 OR S6 OR S7 OR S8	55,438
10	TI ( (memory OR dementia OR alzheimer* OR forget*) N3 (clinic OR clinics OR consult* OR centre* OR center* OR facilit* OR unit OR units OR institute OR institutes OR "primary care" OR service* OR diagnos* OR exam*)) OR AB ( (memory OR dementia OR alzheimer* OR forget*) N3 (clinic OR clinics OR consult* OR centre* OR center* OR facilit* OR unit OR units OR institute OR institutes OR "primary care" OR service* OR diagnos* OR exam*))	11,409
11	S9 AND S10	102
MEDLINE (Ovid)—Search conducted December 2019 Ovid MEDLINE(R) Epub Ahead of Print, In‐Process & Other Non‐Indexed Citations, Ovid MEDLINE(R) Daily, Ovid MEDLINE and Versions(R) 1946 to March 21 2018		
1	exp Nurse Practitioners/	17,534
2	Advanced Practice Nursing/	1597
3	nurse‐led.ab,ti.	3,485
4	"nurse practitioner*".ab,ti.	11,258
5	"nurs* prescri*".ab,ti.	718
6	"advanced nurs* practice*".ab,ti.	502
7	"advanced practice* nurs*".ab,ti.	2,901
8	1 OR 2 OR 3 OR 4 OR 5 OR 6 OR 7	28,593
8	((memory or dementia or alzheimer* or forget*) adj3 (clinic or clinics or consult* or centre* or center* or facilit* or unit or units or institute or institutes or "primary care" or service* or diagnos* or exam*)).ab,ti.	24,117
9	7 AND 8	52
EMBASE (Elsevier)—Search conducted December 2019		
1	'nurse practitioner'/exp	24,973
2	'advanced practice nursing'/exp	1899
3	'nurse practitioner':ab,ti	7,395
4	'nurse‐led':ab,ti	5,855
5	'advanced practice* nurs*':ab,ti	3,363
6	'advanced nurs* practice*':ab,ti	525
7	'nurs* prescri*':ab,ti	804
8	#1 OR #2 OR #3 OR #4 OR #5 OR #6 OR #7	35,838
9	(memory OR dementia OR alzheimer* OR forget*) NEAR/3 (clinic OR clinics OR consult* OR centre* OR center* OR facilit* OR unit OR units OR institute OR institutes OR 'primary care' OR service* OR diagnos* OR exam*)	92,606
10	#8 AND #9	133

Articles focused on nurse‐led memory clinics in dementia care were considered for inclusion if they met the following criteria: (a) published in a peer‐reviewed journal; (b) either a primary study (including qualitative, quantitative and mixed methods), a review, or a descriptive report (professional/clinical articles or cases); (c) written in the English language; and (d) met the definition of “nurse‐led memory clinic” previously described. There were no geographical or publication timeframe limitations imposed.

### 2.4 Search outcome

**FIGURE 1:**
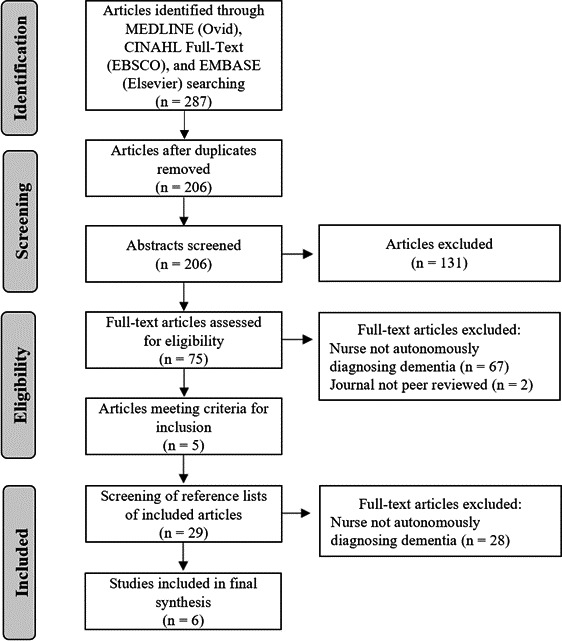
Flowchart of search outcomes (Moher et al., 2009)

All retrieved articles were inputted into Covidence online software, and duplicates were removed (Figure 1). Titles, key words and abstracts were screened by a single reviewer (KL) for their relevance to the research question and inclusion criteria. It should be noted that for articles to be included, they needed to clearly convey the nurse was autonomously diagnosing dementia. Many studies reviewed discussed nurse prescribing, but they did not give enough details to determine whether nurses were also making an autonomous diagnosis or whether the diagnosis was predetermined before the prescribing happened. In these cases, the articles were not included based on missing information. Studies that were questionable for inclusion were reviewed by a second reviewer (*SD*).

Of the 206 retrieved articles, 75 were identified for full review. These articles were read and evaluated against the inclusion criteria. Five articles were included in the final review, and data were then extracted (Clibbens et al., 2019; Hain et al., 2011; McInally, 2015; Minstrell et al., 2015; Stirling et al., 2016). One additional article was added from screening the reference lists of included studies (Hansen et al., 2017). The final number of included papers was six (Figure 1).

### 2.5 Quality appraisal

Due to the methodological heterogeneity of the included articles (e.g. no randomized control trials, no systematic reviews, no inferential statistics) and the descriptive nature of this review, a *formal* quality appraisal or risk of bias assessment could not be conducted. However, quality and risk of bias was considered and commented on for each article (Garritty et al., 2020). Table 2 summarizes the articles included in this review, including the following: type of article, purpose and quality/limitations. As suggested by Grant and Booth (2009), to accommodate for this, additional time was devoted to developing the research questions, synthesizing and exploring the data and reflecting on overall limitations to help counterbalance the lack of a formal quality appraisal in a rapid review. The decision to not reject articles based solely on hierarchical standards of quality is supported by Pawson (2007), where he advocates there are often “nuggets of wisdom in methodologically weak studies” and an appraisal tool should be secondary to the explanatory pursuit of the question one is trying to answer (p. 127). In light of the descriptive nature of the questions in this review, and the limited peer‐reviewed literature on this topic, the authors included all articles that met the inclusion criteria, while acknowledging their limitations and weakness.

**TABLE 2:** Article summary

Article	Type of Article	Purpose	Quality/Limitations
Clibbens et al. (2019)	Descriptive Report	To describe how APN roles were developed and implemented across one National Health Service (NHS) trust to improve the diagnostic pathway for people/carers referred to memory services	Article did not state if services offered in community or hospital; however, website suggests assessment can be done in‐home or in‐clinic. Outcomes reported without any methodological details, just that they came from service users, carer and referrer feedback
Hain et al. (2011)	Descriptive Report	To describe a unique model of care delivery and present a case example illustrating a comprehensive diagnostic evaluation provided at a memory disorder centre	Outcomes stated appeared to be based on anecdotal experiences within the clinic
Hansen et al. (2017)	Formative Program Evaluation	To describe the development and evaluation of a nurse practitioner‐led interprofessional geriatric outpatient clinic called “Inter‐D Clinic”	Lack of standardized outcome measures were reported as a limitation due to the retrospective and observational nature of the evaluation. Low response rate on surveys: patient/caregiver (*N* = 10), PCPs (12); may not be representative of 293 patients seen. Key areas identified for transferability to other locations would be expertise of team members (i.e. comprehensive knowledge of systems and supports) and access to key medical resources, including laboratory and imaging services, pharmacist, physician for restricted medications
McInally (2015)	Case Study	To review and evaluate the effectiveness of a nurse‐led mental health clinic for older adults with a focus on the nurse as a prescriber of ‘memory drugs’	Qualitative feedback was collected from GPs only. No formal evaluation was conducted with patients or carers. Feedback was collected at random and some outcomes reported were anecdotal (e.g. more cost effective, yet nowhere was this analysis shown). Accuracy of diagnosis was compared with prevalence reported by the Alzheimer's society, not with control.
Minstrell et al. (2015)	Case Study	To identify the demographics, assessment scores and diagnostic profiles of those attending an open referral nurse‐led memory clinic (NLMC) and to assess how it differs from other memory clinic profiles	Descriptive statistics for demographics, assessments and diagnoses were compared to other quantitative studies; however due to variability between studies, no quantitative analysis was done. This also made it difficult to determine the contribution of each process towards the outcomes identified. Sample size was also relatively low. Results are descriptive patterns and thus did not report statistical significance
Stirling et al. (2016)	Qualitative Study	To report individuals' experiences after attending a Nurse Practitioner run memory clinic, including clients' experiences of using the clinic; how participation affected their life; and how clinic information impacted behaviour change or understanding	Good congruence between methodology and aim of research, methods used and interpretation of results. No mention of philosophical perspective of researcher. Participant's voices represented well. Limited by small number of participants (13) in one memory clinic, so cannot be generalized

### 2.6 Data abstraction

One reviewer carried out the data extraction (KL) using a data collection form to support the search strategy. In keeping with the guidance of PRISMA (Moher et al., 2009), a data collection form was created to identify variables needed to answer the review questions. This form was created by combining aspects of a quantitative framework called PICO—Problem/Population, Intervention, Comparison and Outcome (Huang et al., 2006), a qualitative tool called SPIDER‐Sample, Phenomenon of Interest, Design, Evaluation outcomes, Research type (Cooke et al., 2012) and input from colleagues. This structured approach ensured relevant data would not be overlooked, was transparent and uniform and supported the compilation of consistent information from a variety of study designs. Data collection included the following: (1) study design/research type; (2) study purpose; (3) location where research was conducted (i.e. country); (4) setting of the memory clinic (e.g. hospital‐based, community); (5) sample/types of patients seen in the memory clinic; (6) additional members of the care team; (7) role of the APN in the clinic; (8) credentials/training of the APN; (9) intervention/details of service provided by the memory clinic; (10) outcomes of the study; and (11) miscellaneous notes (e.g. study limitations, strengths, noteworthy information). Reference and publication information about each study were also collected (e.g. authors, publication date, title, journal).

### 2.7 Synthesis

Due to the descriptive nature of the review questions, methodological heterogeneity among each included study and the low quality of quantitative evidence to statistically evaluate outcomes, the extracted data were synthesized narratively in table format for comparison. This textual approach allowed for the pragmatic blending of evidence to shed light on the descriptive research questions being asked, such as the structures and functions of NLMC; roles and credentials of nurses leading NLMC; and stated outcomes from each article reviewed.

## 3 RESULTS

### 3.1 Study selection and characteristics

A PRISMA flow diagram illustrating the screening and selection of studies for inclusion of this rapid review is presented in Figure 1. The review comprised six articles in total, originating from the United States (Hain et al., 2011), Canada (Hansen et al., 2017), United Kingdom (Clibbens et al., 2019; McInally, 2015) and Australia (Minstrell et al., 2015; Stirling et al., 2016). These articles were published between 2011–2019 and included two case studies (Scotland and Australia; McInally, 2015; Minstrell et al., 2015); two descriptive reports (United States and England; Clibbens et al., 2019; Hain et al., 2011); one qualitative study (Australia; Stirling et al., 2016); and one formative programme evaluation (Canada; Hansen et al., 2017). The purpose of each manuscript varied, ranging from describing the development and model of care used in a NLMC (Clibbens et al., 2019; Hain et al., 2011; Hansen et al., 2017), evaluating the effectiveness of a NLMC (Hansen et al., 2017; McInally, 2015), identifying patient profiles of those that may use a NLMC (Minstrell et al., 2015), to reporting individual patient and caregiver experiences with a NLMC (Stirling et al., 2016).

### 3.2 Structure

The settings described in each article for NLMC included the following: community (Clibbens et al., 2019; Minstrell et al., 2015; Stirling et al., 2016), university campus (Hain et al., 2011), hospital (Hansen et al., 2017) and general practitioner's office (McInally, 2015). Articles included participants/patients with concerns regarding cognitive decline and memory functioning (Clibbens et al., 2019; Hain et al., 2011; Hansen et al., 2017; McInally, 2015; Stirling et al., 2016) or those already attending a NLMC (Minstrell et al., 2015; Stirling et al., 2016). Of the six NLMC described, four required a physician referral (Clibbens et al., 2019; Hansen et al., 2017; McInally, 2015; Stirling et al., 2016) and two had an open referral system where individuals with memory concerns could refer themselves, or be referred by another community agency (Hain et al., 2011; Minstrell et al., 2015). The NLMCs included multidisciplinary teams, which could include psychologists, social workers, occupational therapists and/or physiotherapists (Clibbens et al., 2019; Hain et al., 2011; Hansen et al., 2017); teams consisting of just the nurse and consulting physician (Minstrell et al., 2015; Stirling et al., 2016); and a team with a nurse and doctor with ad‐hoc referrals to allied health care providers (e.g. psychologist) as needed (McInally, 2015).

### 3.3 Functions, roles and credentials

The services offered by all NLMC included assessment, diagnosis and treatment/postdiagnostic care, which could include prescribing, developing care plans, referrals for services and follow‐up care. The nurse had a lead role in these functions in each NLMC. Credentials of these nurses included a community psychiatric nurse/non‐medical prescriber (McInally, 2015) and APNs (Clibbens et al., 2019; Hain et al., 2011; Hansen et al., 2017; Minstrell et al., 2015; Stirling et al., 2016). In four of the articles (clinics located in Canada, USA and Australia), the APNs were specified as nurse practitioners (Hain et al., 2011; Hansen et al., 2017; Minstrell et al., 2015; Stirling et al., 2016). One clinic in England employed a mental health nurse practicing as an APN who was an independent prescriber with a specialized postgraduate certificate in dementia (which included preceptorship and supervisory processes; Clibbens et al., 2019). Another clinic in Scotland, situated in a small community, employed a community practice nurse with over 20 years of experience with a non‐medical prescribing licence (McInally, 2015).

### 3.4 Outcomes

The main stated conclusions described in the six articles included the following: NLMC enhanced access to diagnosis and care (Clibbens et al., 2019; McInally, 2015; Minstrell et al., 2015; Stirling et al., 2016); NLMC were an effective service delivery model (Clibbens et al., 2019; Hain et al., 2011; McInally, 2015); NLMC offered high quality of care delivery (Clibbens et al., 2019; Hansen et al., 2017; Stirling et al., 2016); NLMC had high stakeholder satisfaction with patient/caregivers (Hain et al., 2011; Hansen et al., 2017; Stirling et al., 2016) and primary care providers (Hansen et al., 2017; McInally, 2015); and NLMC enhanced the role of nurses (Clibbens et al., 2019). Both case studies also supported the effectiveness of nurses diagnosing dementia (McInally, 2015; Minstrell et al., 2015).

A result summary table (see Table 3) features additional details for each NLMC in terms of functions (i.e. clinic details, details of assessment) and roles (i.e. the role of the lead nurse, practice guidelines/policies).

**TABLE 3:** Results summary

Article	Functions		Roles	
	Clinic details	Details of assessment	Role of lead nurse	Practice guidelines/protocols/policies
Clibbens et al., (2019)	Not available	Not available	Assessment, diagnosis and treatment, including requests for CT scans and MRI brain scans	Practices within clear evidence‐based dementia pathways that specify their roles and functions
Hain et al., (2011)	Not available	3 visits focused on history/physical, neuropsychological assessment, comprehensive counselling and care coordination	History/physical, screening tests, laboratory/neuroimaging studies, disclosure of diagnosis, patient/family comprehensive counselling and care coordination (including interactive dialogue regarding interventions, education and resources), evaluation report to primary care provider	Practices under physician‐approved protocols as required by the State; however, functions as independent member of the interdisciplinary diagnostic team
Hansen et al., (2017)	1 day/week	Initial assessment: 2.5 hr	Medical assessments, order investigations, provide diagnoses, offer/arrange postdiagnostic care, make referrals to other medical specialists as required	Licensed to work in Ontario, Canada
McInally (2015)	4‐hr clinic/month	Each visit is scheduled for 45 min	Initial assessment, order CT scans, refer to a psychologist if needed to confirm diagnosis, determine treatment plan, provide prescriptions (or direct GP to write prescription), follow‐up, refer to other agencies, provide ongoing support, enter assessment findings/investigations/treatment plan into electronic medical record	Non‐medical prescribers can assess, diagnose and prescribe independently
Minstrell et al., (2015)	1 day/week	2 visits. Initial assessment: 1.5–2.5 hr Second visit: personalized care plan	Medical history and physical assessment (including standardized assessment tools), separate interviews with both the patient and accompanying person, order blood tests and imaging when clinically indicated, prepare personalized care plan, creates summary report detailing diagnosis, recommendations and plan for follow‐up	Works within a collaborative agreement with the state health department. NPs diagnoses are discussed with an old age psychiatrist (post hoc) to enable the specialist to commence drug treatment (e.g. cholinesterase inhibitors), or order brain scans if necessitated (as NPs across Australia are not allowed to initiate this type of treatment)
Stirling et al., (2016)	1 day/week	Initial assessment: 2–2.5 hr	Medical/cognitive assessment, provide diagnosis, offer supportive information and makes referrals for services	Works with collaborative support from an Old Age Psychiatrist

Abbreviations: CT scan, computerized tomography; GP, general medical practitioner; MRI, magnetic resonance imaging;NP, nurse practitioner.

## 4 DISCUSSION

The goal of this review was to explore the existing peer‐reviewed evidence to illuminate the structures, functions and outcomes of nurse‐led memory clinics, and the nursing roles and credentials of nurses leading memory clinics to inform discussions about interventions and innovations to improve the diagnosis and treatment of dementia; and identify areas for future research. The limited number of articles identified not only speaks to this newer and evolving role for nurses, but also illustrates the paucity of evidence that specifically examines the impact of nurses diagnosing dementia and leading postdiagnostic care. The limited peer‐reviewed publications on this topic may not necessarily reflect a lack of nurses working autonomously in dementia care, but rather a gap in knowledge translation of what is happening on the front lines of health care and what gets published. As revealed by Song et al. (2014), many health‐related studies (median of 85%) go unpublished due to factors such as time constraints or low priority. It does appear NLMC, as defined for this review, are becoming more commonplace (Duffin, 2009; Reves et al., 2018); however, the current peer‐reviewed literature does not reflect this.

The structure of the nurse‐led memory clinics ranged in their physical location from clinics offered in an institution to clinics offered in the community. All the clinics appeared to see similar clients, consisting of individuals that had concerns about memory function or cognitive decline. Most patients had non‐complex dementia. More challenging cases would be referred to a specialist for assessment, which is what happens in general medical practitioner run memory clinics (Lee et al., 2014, 2019; Stone et al., 2019).

Access to the clinics varied between requiring a referral by their primary care provider and allowing individuals with concerns to refer themselves or be referred by another community agency. Minstrell et al. (2015) suggest open referral policies that allow individuals to self‐refer to a memory clinic when they have concerns about their own cognitive function can remove obstacles that might delay access to early diagnosis of dementia. From the operational details provided, it appeared most clinics operated one day per week and initial assessments ranged from one–three visits and could last between 45 min–2.5 hr. All six clinics had the nurse leading the diagnosis and care planning for individuals with dementia; however, all nurses had some form of medical support, either in a consultatory or collaborative structure, with a doctor (i.e. GP, geriatrician or old age psychiatrist). The structure of the NLMC reviewed is quite like more traditional memory clinics described in the literature (Braekhus et al., 2011; Jolley et al., 2006; Jolley & Moniz‐Cook, 2009; Van der Cammen et al., 1987). Jolley et al. (2006) identified the essential attributes of a memory clinic, which includes dedicated time and space, a core team, links to other agencies including the Alzheimer's society and expertise of other disciplines. The latter attribute, expertise of other disciplines, varied the most among the clinics, where some worked in a multidisciplinary team (Clibbens et al., 2019; Hain et al., 2011; Hansen et al., 2017) and others had only a team consisting of the nurse and specialist (Minstrell et al., 2015; Stirling et al., 2016). In these situations, it is unknown if this was in fact the case, or if other team members were just not mentioned in the article.

The main functions of the NLMC appeared similar across all clinics, irrespective of location, including assessment, diagnosis and treatment/postdiagnostic care. These services did not appear to differ from the essential activities identified by Jolley et al. (2006), or from those in traditional memory clinics that are led by specialized medical staff (Braekhus et al., 2011; Jolley et al., 2006; Jolley & Moniz‐Cook, 2009; Van der Cammen et al., 1987), including assessment/investigation, diagnosis (including differential diagnosis), communication of findings with patients/caregivers, connecting with other community agencies, providing treatment, monitoring progress, patient/caregiver education and health promotion. Even when compared with another primary care‐based memory clinic models (Dodd et al., 2016; Lee et al., 2014; Wells & Smith, 2017), the central functions of assessment, diagnosis, treatment and postdiagnostic care were similar to what was described in the NLMC reviewed. One essential area mentioned by Jolley et al. (2006) that was not directly commented on in the articles included in this review was research and auditing; however, one might assume research and auditing, to some degree, were being implemented since all six of these clinics published articles on their NLMC.

The roles of the nurses leading the care in each of the memory clinics were comparable. Nurses were involved with leading the assessments (e.g. medical examination, cognitive assessment), ordering investigations (e.g. bloodwork, CT/MRI as required), diagnosing, prescribing medications and developing a plan of care (including resources and referrals). It should be noted, however, that in Australia only medical specialists can prescribe for cholinesterase inhibitors and order MRI testing (Minstrell et al., 2015). In these situations, the nurse practitioner would have to consult with an old age psychiatrist to get these test/prescriptions ordered; yet, they had full autonomy to assess, diagnose and prescribe other medications for the individuals they cared for.

The credentials of nurses with a lead role in NLMC were similar in most cases. The majority were APNs (Clibbens et al., 2019; Hain et al., 2011; Hansen et al., 2017; Minstrell et al., 2015; Stirling et al., 2016), and one was a community psychiatric nurse and non‐medical prescriber (McInally, 2015). Nurses licensed as independent prescribers in the United Kingdom are able to assess, diagnose and independently prescribe medications and some controlled substances (Courtenay et al., 2011). In contrast, nurses that are supplementary prescribers can only prescribe medications as set out in a clinical management plan after an assessment and diagnosis is made by a physician. While the specific details for the full scope of practice and licensure of the nurses in each clinic were not always provided, the numerous nursing titles used for the nurses working in similar NLMC highlight the confusion that can be created for the various roles and skills of APNs (Bishop, 2014). Through the development of an APN consensus model, the National Council of State Boards of Nursing (2020) advocate the need to standardize APN regulations to improve awareness, understanding, public protection and accountability. While this initiative is being implemented across the United States, this alignment in nursing roles would be beneficial internationally to address similar issues and promote the value APNs offer; specifically, in improving dementia care.

Overall, there was low quality of evidence to evaluate outcomes. The six articles identified for this review were either self‐reported, had small samples sizes, were limited in the type of quantitative data collected and analysed, or lacked standardized outcome measures. The level of evidence and lack of experimental trials in other types of nurse practitioner‐led clinics have been identified in various systematic reviews whereby firm conclusions could not be drawn and more research was warranted (Leonard, 2006; Shah & Deswal, 2016; Whiteford et al., 2016). Mullins et al. (2016) also share this view and stress the importance of evidence to support nurses practicing to their full scope, and to demonstrate the impact they can make in improving patient outcomes with the growing population of older adults. While the breadth and depth of studies included in this review, or lack thereof, cannot provide a definitive answer to the impact of NLMC, the prominent themes from the stated outcomes in each article reviewed do suggest NLMC are an effective service delivery model to improve access for dementia diagnosis and treatment; offer quality care and reliable diagnosis; and have high levels of stakeholder satisfaction. Similar results have been reported with other nurse‐led clinics (i.e. clinics being led by Registered Nurses, specialist nurses and/or nurse practitioners, with varying degrees of autonomy and responsibility) in areas such as orthopaedics (Flynn, 2005), kidney disease (Coleman et al., 2017), community medicine (Kant et al., 2018) and arthritis care (Garner et al., 2017). This review highlights the need for research with thorough methodologies, focused on outcomes, to inform evidence‐based decisions.

This review provides insight into how current NLMC are structured and how they function, including the roles and credentials of the nurses leading the memory clinic processes to inform nursing practice. Additional implications for practice based on the outcomes reported suggest APNs can be a potential solution for improving dementia care. It is expected that as the role of APNs in memory clinics continue to expand and more high‐quality research is conducted and published, the value of APNs in dementia care will be substantiated. However, in the interim, only anecdotal conclusions, albeit very promising, can be made.

Future research is needed to address both the quality and quantity of the current evidence for nurse‐led memory clinics, and how this novel approach can contribute to improving dementia care. Randomized control trials or research employing other rigorous methodologies are needed to explore quality of care/postdiagnostic care, timeliness of care, cost‐effectiveness, efficiencies, stakeholder satisfaction and reliability of diagnosis. This would greatly contribute to the current deficiency in knowledge in this area and help to inform innovative approaches to address both the current and future challenges faced in caring for those with, or at risk for, dementia. Additional topics that would also enhance our understanding in this area would include exploration of the barriers to implementing such a nurse‐led memory clinic model, and what health disciplines are most critical to offering optimal care.

A systematic process was followed to conduct this rapid review; however, there were still several limitations. This rapid review only used three databases to search for peer‐reviewed articles; therefore, some articles may have been missed for inclusion in this review. Due to time and resource limitations, articles were reviewed by only one reviewer, except for studies deemed questionable for inclusion by that reviewer. Having a second reviewer independently review all the articles may have resulted in additional articles for inclusion. The heterogeneity of articles reviewed and the lack of systematic review evidence or randomized control trials was a limitation for doing a formal quality appraisal as part of the review process. This heterogeneity and the various levels of details described in each article also made it challenging to paint a holistic picture of the structures and functions of each clinic for comparison.

## 5 CONCLUSIONS

The prevalence of dementia is on the rise. Maintaining the status quo in how we currently diagnose and treat dementia could be troublesome, not only for those with dementia and their caregivers, but for the healthcare system and our communities as a whole. Innovative approaches are needed today and for the future, to address this issue and to ensure individuals with dementia can access timely diagnostic and postdiagnostic care. Nurses with advanced training (i.e. NPs, non‐medical prescribers) have the skill set to offer a viable solution to improve access to diagnosis and needed care for those with, or at risk, for dementia. The paucity of published peer‐reviewed literature on NLMC makes it difficult to come to any firm conclusions; however, the existing evidence and the trends identified in the literature suggest NLMC could be an innovative solution to enhancing dementia care and warrants further exploration.

## CONFLICT OF INTEREST

No conflict of interest has been declared by the author(s).

## AUTHOR CONTRIBUTIONS

Both authors have agreed on the final version of the manuscript and quality for authorship by meeting all criteria of the journal's authorship policy found at https://onlinelibrary.wiley.com/page/journal/13652648/homepage/forauthors.html#editorial, including: Substantial contribution to conception and design (KL and SD), or acquisition of data (KL), or analysis and interpretation of data (KL). Drafting the manuscript (KL) or revising it critically for important intellectual content (KL and SD). Participating sufficiently in the work to take public responsibility for appropriate portions of the content (KL and SD). Being accountable for all aspects of the work in ensuring that questions related to the accuracy or integrity of any part of the work are appropriately investigated and resolved (KL and *SD*).

## ETHICAL APPROVAL

Research ethics committee approval was not required for this review.

## PATIENT CONSENT STATEMENT

This review did not need to seek patient consent.

## Data Availability

The data that support the findings of this review are noted in the body of the article and identified in the reference list.
